# Supplementary material for: Biologically relevant integration of transcriptomics profiles from cancer cell lines, patient-derived xenografts, and clinical tumors using deep learning
Source: Sci Adv. 2025 Jan 17;11(3):eadn5596. doi: 10.1126/sciadv.adn5596 (PMC11740957; doi:10.1126/sciadv.adn5596)
Supplement: Supplementary file 1 — Supplementary Text Figs. S1 to S11 [file sciadv.adn5596_sm.pdf]

Science Advances
AAAS

Supplementary Materials for

Biologically relevant integration of transcriptomics profiles from cancer cell lines, patient-derived xenografts, and clinical tumors using deep learning

Slavica Dimitrieva *et al.*

Corresponding author: Slavica Dimitrieva, slavica.dimitrieva@novartis.com

Sci. Adv. **11**, eadn5596 (2025)
DOI: 10.1126/sciadv.adn5596

This PDF file includes:

Supplementary Text
Figs. S1 to S11

Benchmarking MOBER's performance relative to other popular batch effect correction methods

We compared MOBER against four widely used batch correction methods: *ComBat* (*21*, *22*), *Harmony* (*34*), *Batch Mean Centering*(*35*) and the *Regress_Out* algorithm as implemented in *scanpy* (*36*). *ComBat* leverages a parametric and non-parametric empirical Bayes approach for correcting the batch effect. *Harmony* corrects for batch effects by computing a low dimensional embedding and altering the embedding of each cell with respect to batches. The *Batch Mean Centering (BMC)* method adjusts data by subtracting the batch-specific mean from each gene expression value, aiming to standardize batch effects across the dataset without the need for external variables. The *Regress_Out* algorithm uses *simple linear regression* (denoted in the plots below as *SLR*) to regress out unwanted sources of variation.

To ensure detailed and unbiased comparison, we evaluated these methods by assessing the proximity of samples from the same indication but different batches following batch correction. In this respect, for each sample we calculated the Euclidean distance within 70-dimensional PCA space to the first nearest neighbor from the same indication but different batch, as well as the average distance to the 25 nearest neighbors from the same indication, but different batch. Smaller distances between samples from the same indication but different batches indicate better alignment.

Fig. S1a-d show the alignment of pre-clinical and clinical tumor transcriptomes using different methods. None of these methods was able to effectively align CCLE and PTX to the TCGA data. Fig S1e-f indicate that, after batch correction, the smallest average distance between samples from the same indication, but different batches is observed with MOBER.

Next, we simulated batch effects in RNA-seq data and created five different datasets (denoted as Dataset1 – Dataset5) with varying levels of confounding factors. To create each of these datasets we started from the TCGA dataset and randomly partitioned it into four non-overlapping partitions (denoted as batch0 – batch3). Prior to this, we excluded TCGA primary sites with fewer than 100 samples to ensure statistical robustness. In the TCGA partitions we introduced gene expression variabilities as follows:

- Technical variability per sample - introduced to simulate individual sample handling differences, equipment variations, or other laboratory-specific factors that could influence the measurements independently of biological conditions. This was done by sampling noise from a normal distribution as follows:
  sample noise level = random.normal(0, random.uniform(0.0, 0.4))
- Random noise on a gene level - small noise was added to expression levels of genes to reflect the stochastic nature of RNA transcription and sequencing, enhancing the complexity of the dataset. This noise was sampled from a uniform distribution as follows:
  noise on gene level = random.uniform(-0.25, 0.25)
- Differential gene expression – we introduced modifications of gene expression levels on both batch level and indication level to simulate the inherent variability between different biological entities (e.g. difference in TME-related genes between cell lines, patient-derived xenografts and human tissues). The modification factor per gene was applied by sampling from a random distribution as follows:
  modification factor per gene = random.uniform(0.25, 4)

We note that in certain studies we might not want to remove batch effects resulting from gene perturbations. However, here we introduce such modifications to simulate the systematic differences between pre-clinical models and clinical tumors, aiming to remove them.

The above variabilities were applied on the TCGA samples to create each of the five datasets as follows:

- **Dataset1**: In the three out of the four TCGA partitions we introduced small technical variability per sample, small random noise on a gene level, and modified the expression of 1000 randomly selected genes by a random factor, as stated above.
- **Dataset2**: Similar to Dataset1, but we additionally introduced gene expression modifications for 1000 randomly selected genes per indication. Modifying genes per indication was done to simulate differences in the TME between different tumor indications.
- **Dataset3**: Same as Dataset1, but instead of 1000 genes we introduced expression modifications to 5000 genes in each of the batches to simulate stronger confounding batch effects.
- **Dataset4**: Similar to Dataset2, we introduced gene expression modifications to 3000 randomly selected genes per batch and 3000 randomly selected genes per indication. The sampled genes per batch and per indication can overlap.
- **Dataset5**: Similar to Dataset2 and Dataset4, we introduced gene expression modifications to 5000 randomly selected genes per batch and 5000 randomly selected genes per indication. The sampled genes per batch and per indication can overlap.

Fig. S2 - S6 show the alignment of the four different batches in each of the simulated datasets respectively, with each of the tested methods. To quantitatively measure the quality of the batch alignments in each of the five simulated datasets, we again computed the average Euclidean distance within 70-dimensional PCA space to the first nearest neighbor from the same indication but different batch for each sample, as well as the average distance to the 25 nearest neighbors from the same indication, but different batch. Fig. S7 demonstrates the superior performance of MOBER in comparison to the other tested methods in aligning the different batches from the 5 simulated datasets.

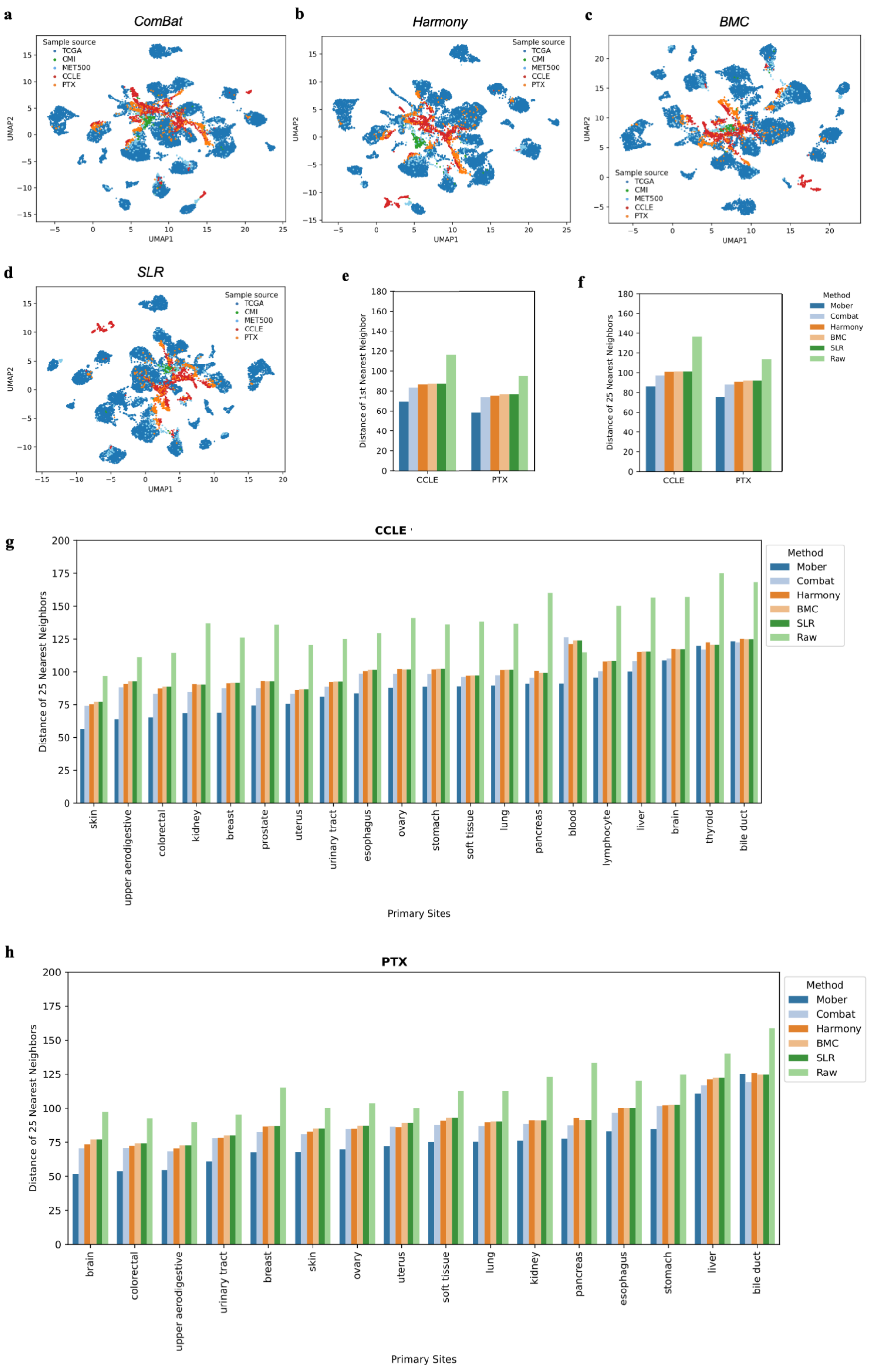

Fig. S1 | Batch effect correction with *ComBat, Harmony, BMC*, and *SLR* in comparison to MOBER. Integration of transcriptional profiles from models and patient tumors using a) *ComBat*, b) *Harmony*, c) *BMC*, and d) *SLR*. The color corresponds to the sample origin. e) and f) Average Euclidean distance within 70-dimensional PCA space to the first nearest neighbor (e) or to the 25 nearest neighbors (f) from the same indication but different batch for each CCLE and PTX sample respectively. g) Average distance of CCLE samples to the 25 TCGA nearest neighbors from the same indication, stratified by tumor type. h) Average distance of PTX samples to the 25 TCGA nearest neighbors from the same indication, stratified by tumor type.

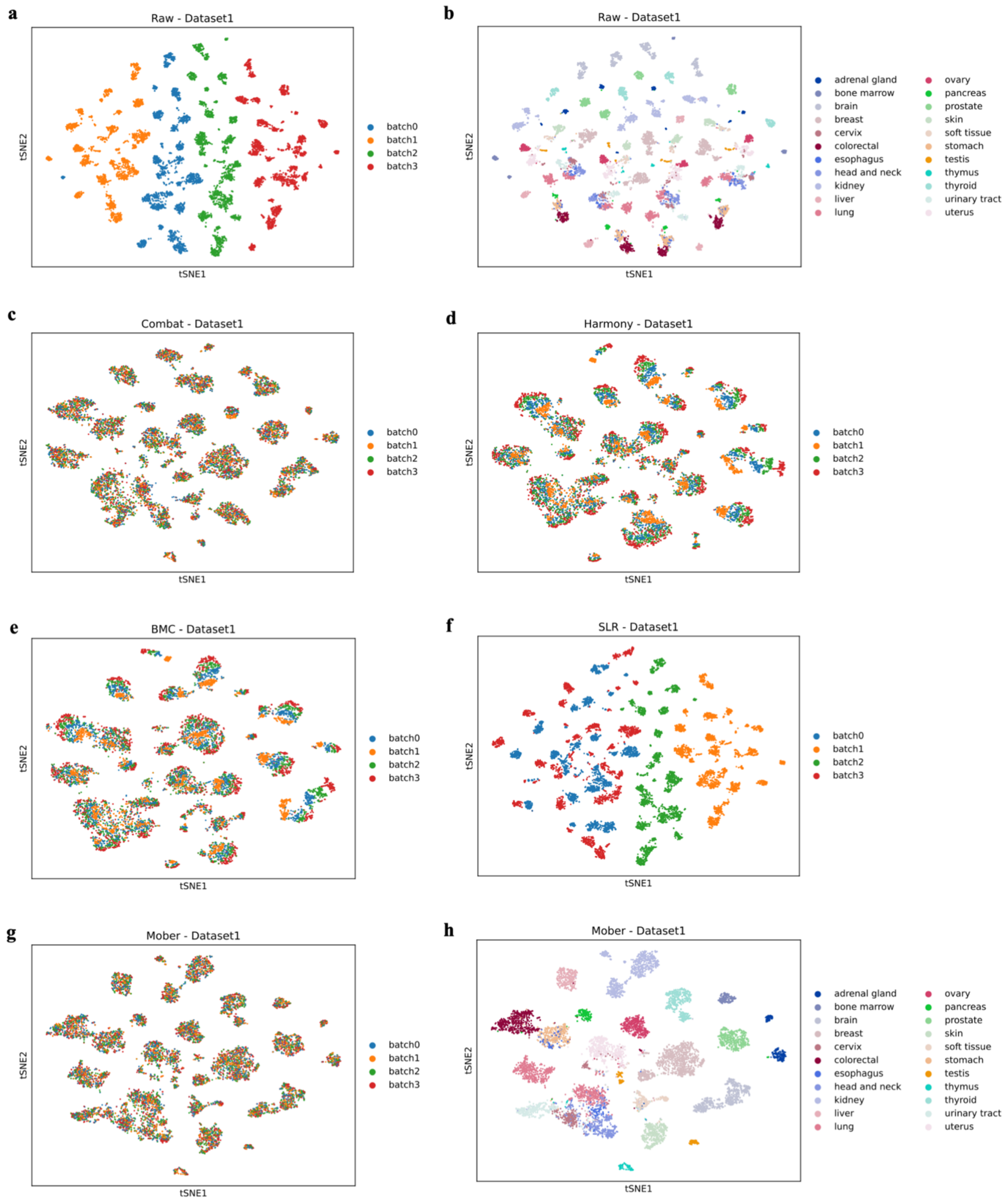

Fig. S2 | Batch effect correction in simulated Dataset1. a) and b) Alignment of samples from the 4 simulated batches without any batch correction. The samples are colored by batch in (a) and by disease type in (b). Batch effect correction with c) *ComBat*, d) *Harmony*, e) *BMC*, f) *SLR* and g) MOBER. The samples are colored by batch. h) Batch effect correction with MOBER like in g), but with samples colored by disease type.

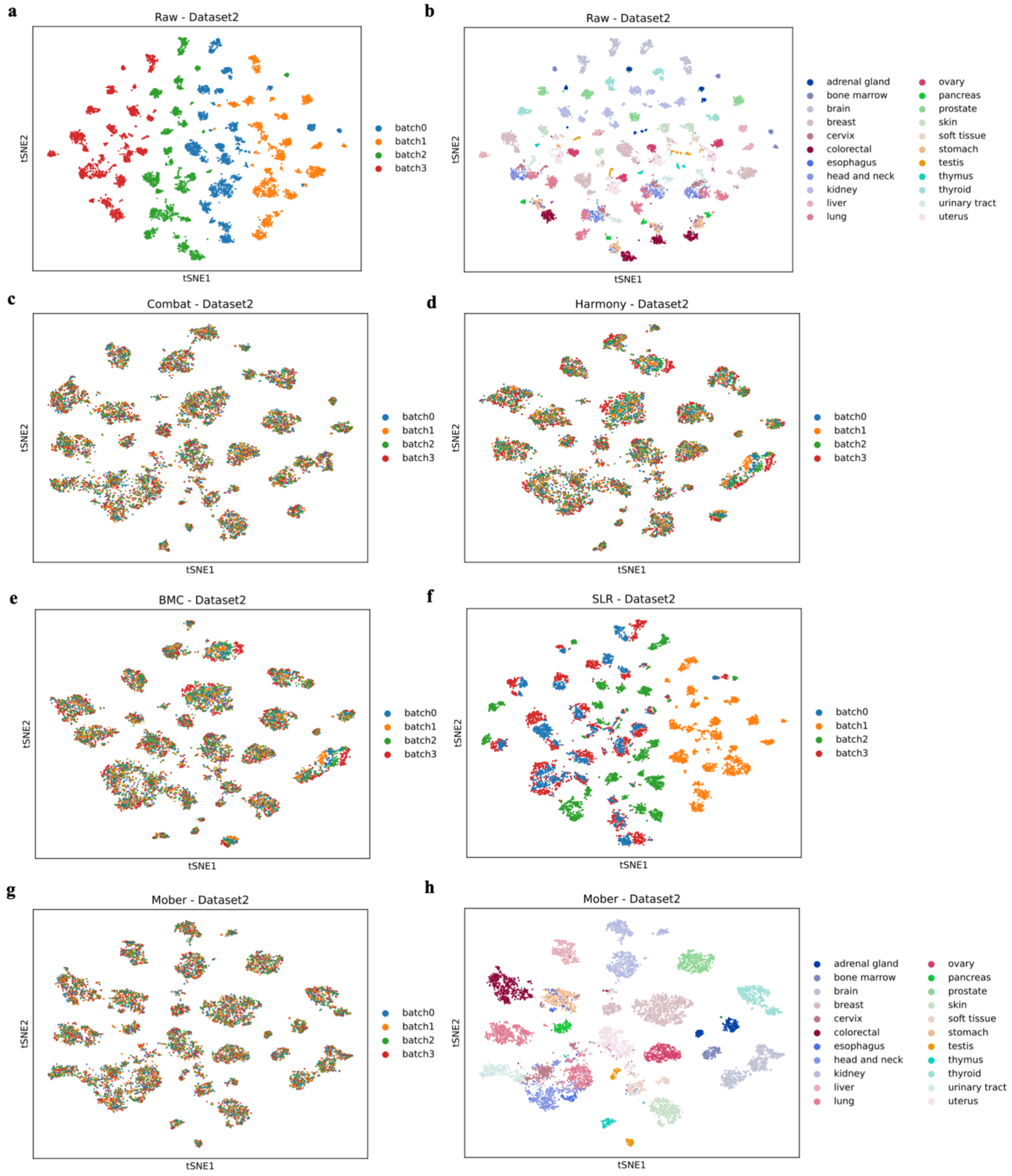

Fig. S3 | Batch effect correction for simulated Dataset2. a) and b) Alignment of samples from the 4 simulated batches without any batch correction. The samples are colored by batch in (a) and by disease type in (b). Batch effect correction with c) *ComBat*, d) *Harmony*, e) *BMC*, f) *SLR* and g) MOBER. The samples are colored by batch. h) Batch effect correction with MOBER like in g), but with samples colored by disease type.

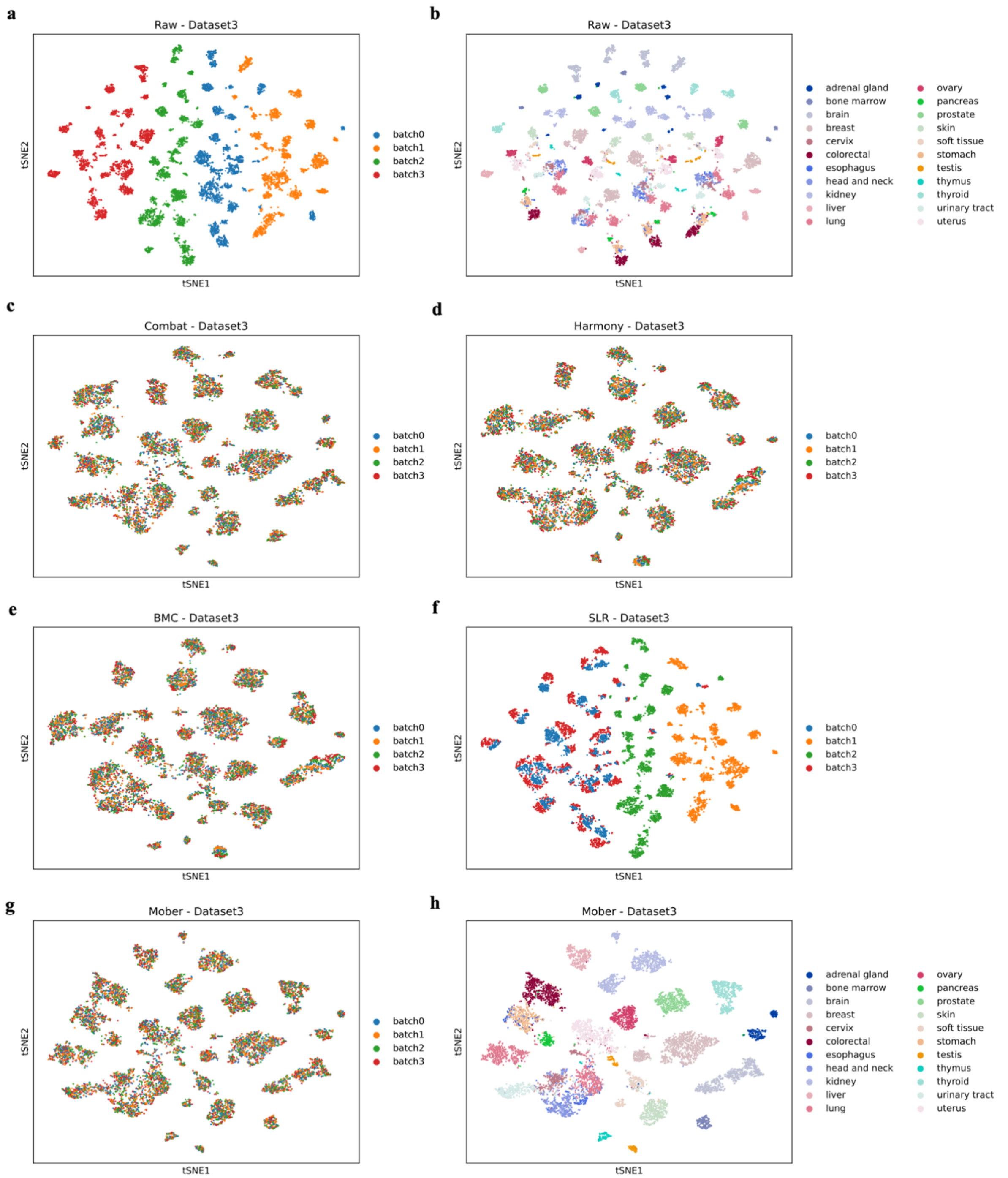

Fig. S4 | Batch effect correction in simulated Dataset3. a) and b) Alignment of samples from the 4 simulated batches without any batch correction. The samples are colored by batch in (a) and by disease type in (b). Batch effect correction with c) *ComBat*, d) *Harmony*, e) *BMC*, f) *SLR* and g) MOBER. The samples are colored by batch. h) Batch effect correction with MOBER like in g), but with samples colored by disease type.

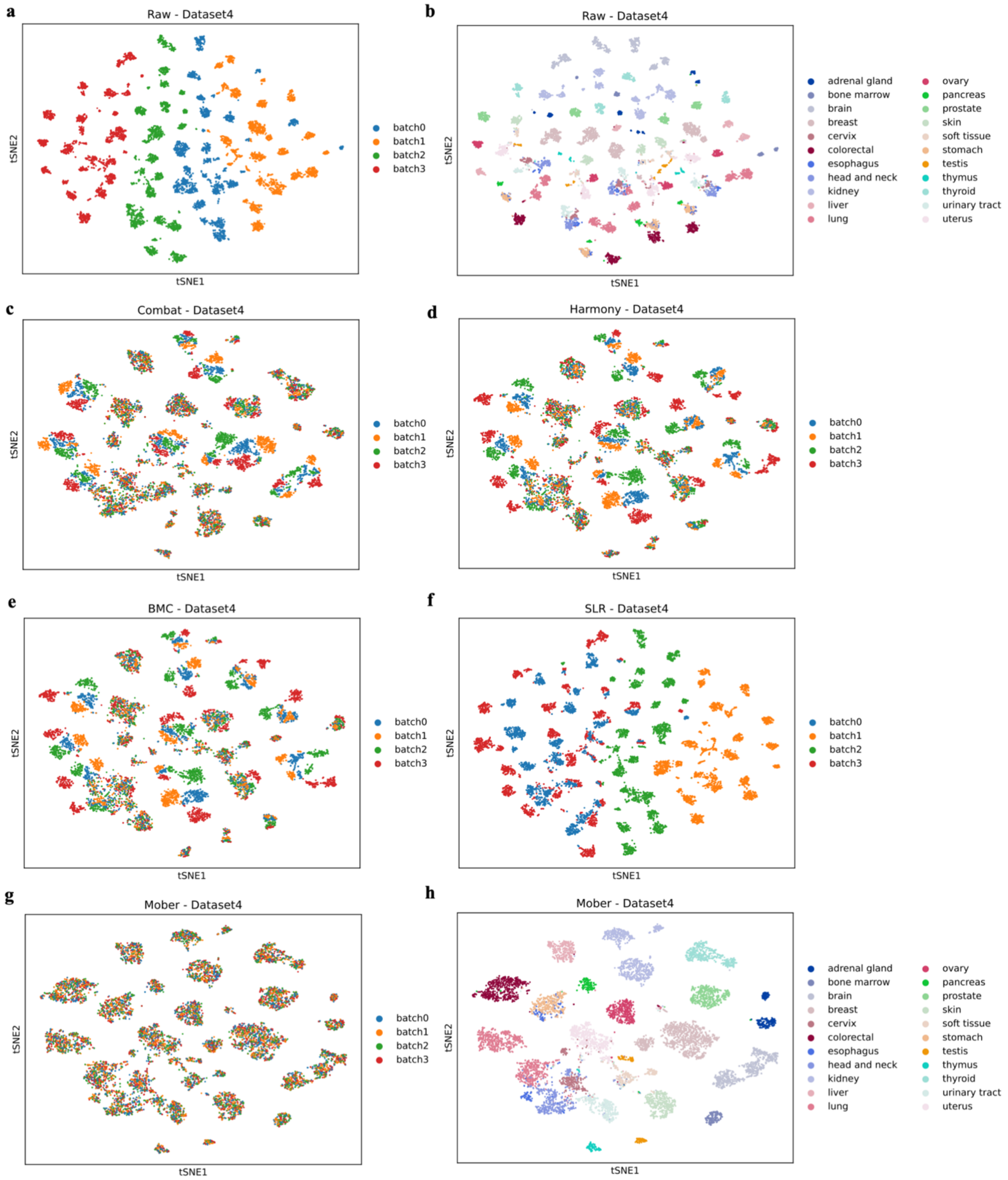

Fig. S5 | Batch effect correction in simulated Dataset4. a) and b) Alignment of samples from the 4 simulated batches without any batch correction. The samples are colored by batch in (a) and by disease type in (b). Batch effect correction with c) *ComBat*, d) *Harmony*, e) *BMC*, f) *SLR* and g) MOBER. The samples are colored by batch. h) Batch effect correction with MOBER like in g), but with samples colored by disease type.

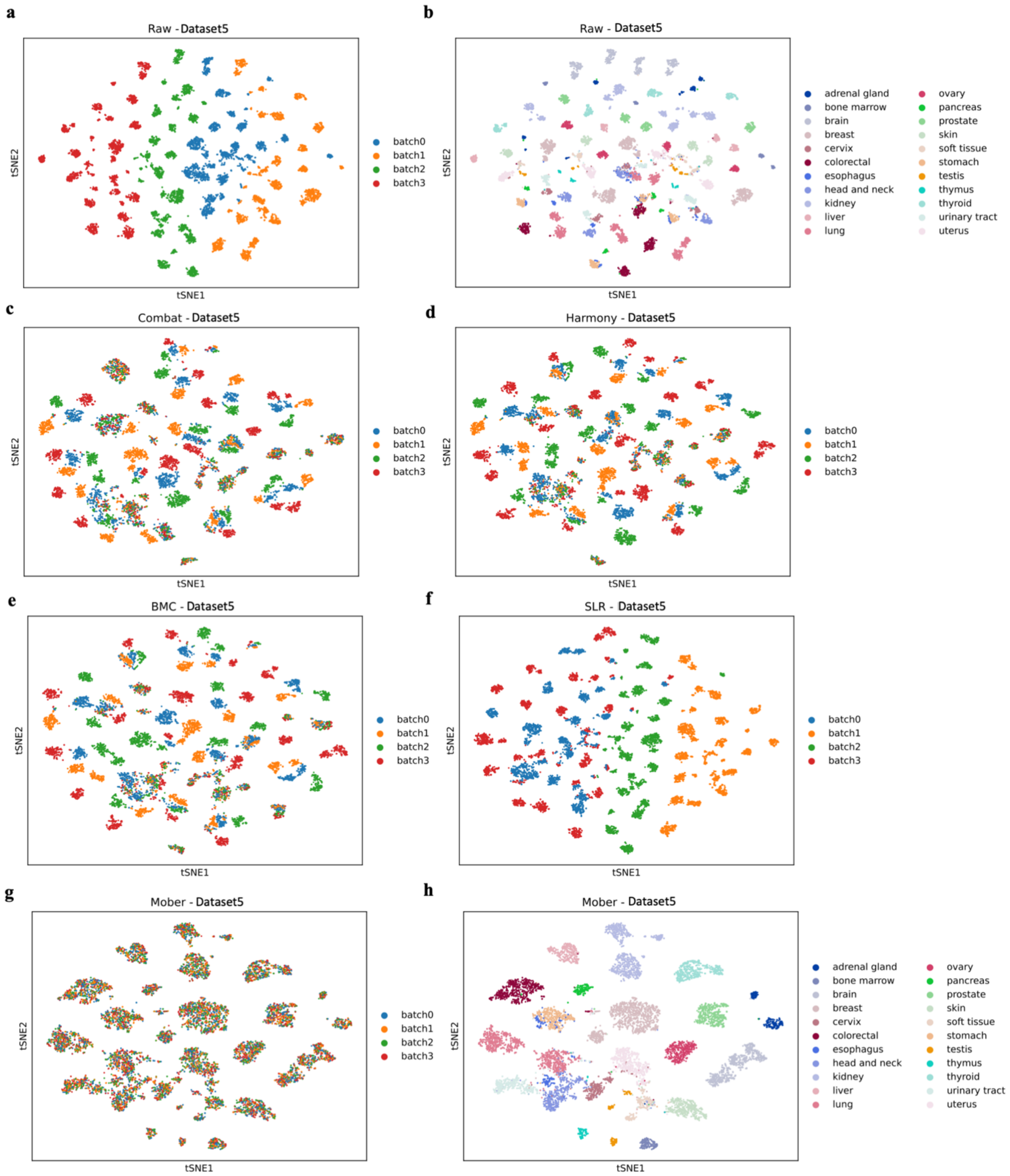

Fig. S6 | Batch effect correction in simulated Dataset5. a) and b) Alignment of samples from the 4 simulated batches without any batch correction. The samples are colored by batch in (a) and by disease type in (b). Batch effect correction with c) *ComBat*, d) *Harmony*, e) *BMC*, f) *SLR* and g) MOBER. The samples are colored by batch. h) Batch effect correction with MOBER like in g), but with samples colored by disease type. This dataset exhibits very strongly pronounced batch effects, yet MOBER could still successfully align the batches and cluster the samples by disease type (h).

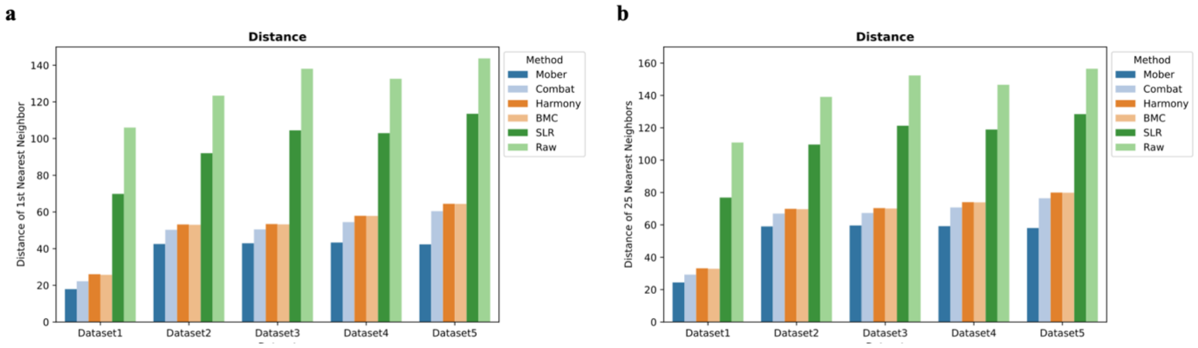

Fig. S7 | Performance on the 5 simulated datasets by different methods. a) Average distance to the first nearest neighbor (a) or to the 25 nearest neighbors (b) from the same indication but different batch for each sample in the 5 simulated datasets. MOBER consistently outperforms the other methods.

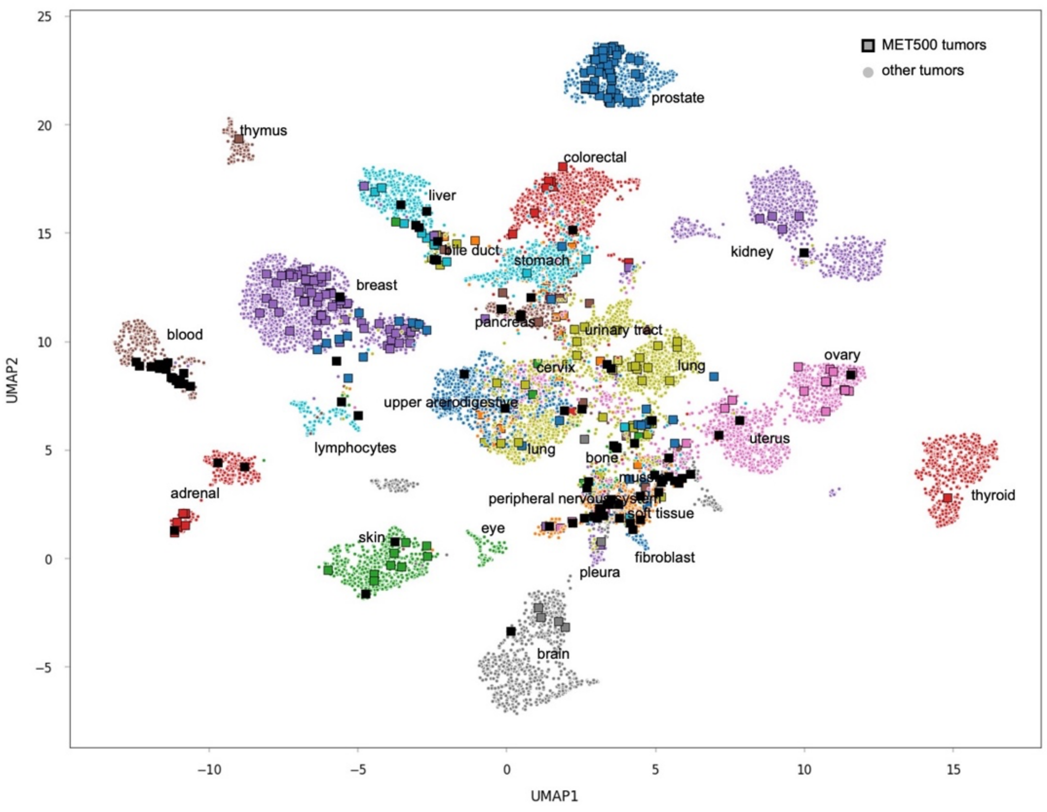

Fig. S8 | Global pan cancer alignment with MOBER, highlighting MET500 metastatic samples. MET500 tumors are shown in squares with color corresponding to the primary site. The samples with unknown primary origin are shown in black. All other tumors (CCLE, PTX, CMI and TCGA) are shown with circles and are colored based on their primary site annotation.

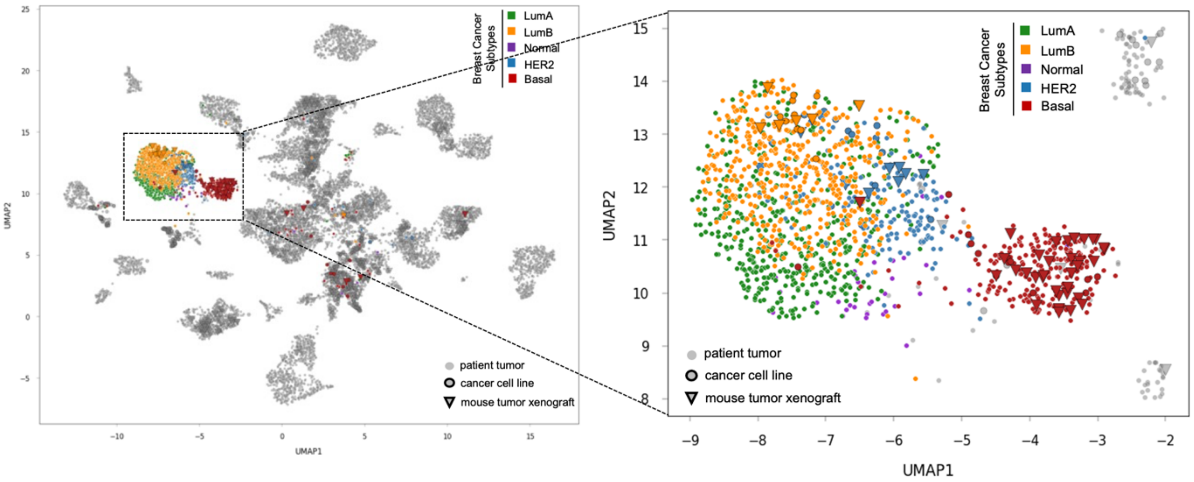

Fig. S9 | Alignment of breast cancer subtypes. UMAP 2D projection of the MOBER-alignment highlighting breast tumor samples: LumA (green), LumB (orange), Normal (purple), HER2 (blue) and Basal (red). All other non-breast tumor samples are in grey.

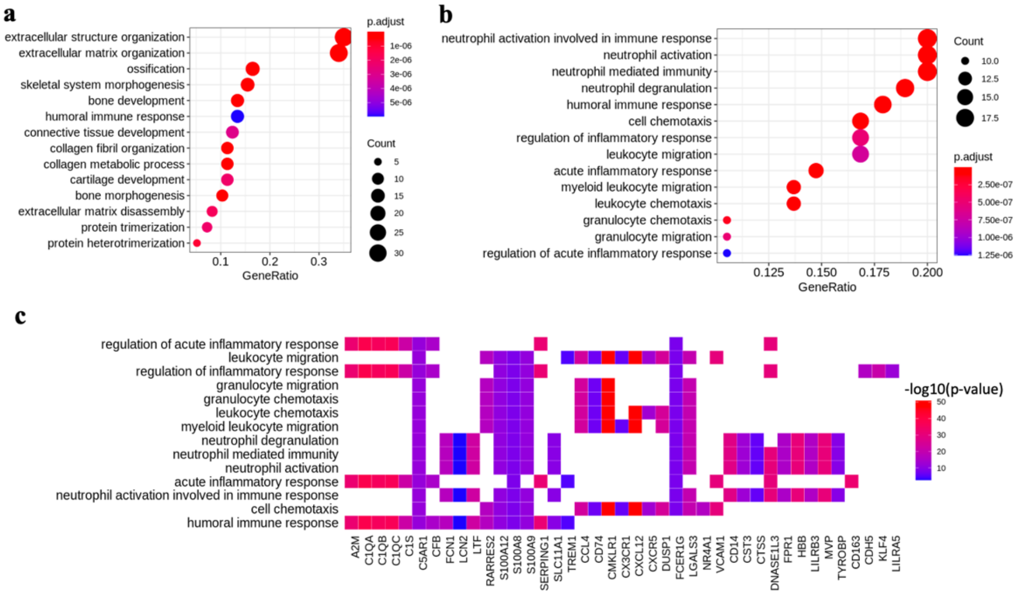

Fig. S10 | Pathway enrichment analysis of the top 100 upregulated genes when transforming CCLEs to TCGA tumors for a) breast primary tumors; b) blood tumors. c) Genes that are most significantly upregulated *in silico* after the alignment of blood CCLEs to blood TCGA tumors (x-axis), along with top enriched biological pathways involving the 100 most upregulated genes (y-axis).

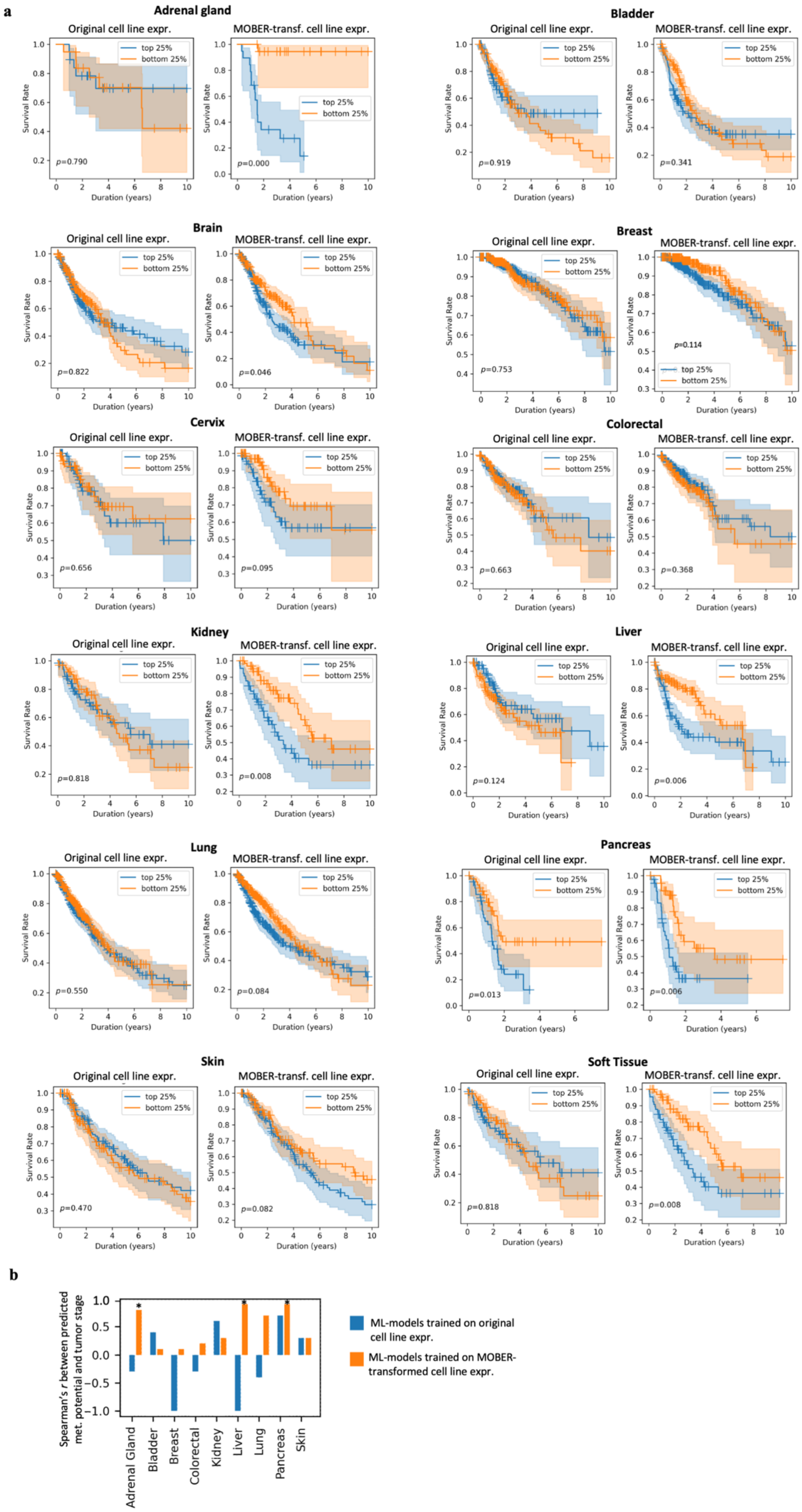

a
Adrenal gland
Original cell line expr.
MOBER-transf. cell line expr.
p=0.790
p=0.000
Bladder
p=0.919
p=0.341
Brain
p=0.822
p=0.046
Breast
p=0.753
p=0.114
Cervix
p=0.656
p=0.095
Colorectal
p=0.663
p=0.368
Kidney
p=0.818
p=0.008
Liver
p=0.124
p=0.006
Lung
p=0.550
p=0.084
Pancreas
p=0.013
p=0.006
Skin
p=0.470
p=0.082
Soft Tissue
p=0.818
p=0.008
top 25%
bottom 25%
Survival Rate
Duration (years)
b
Spearman's r between predicted met. potential and tumor stage
Adrenal Gland
Bladder
Breast
Colorectal
Kidney
Liver
Lung
Pancreas
Skin
ML-models trained on original cell line expr.
ML-models trained on MOBER-transformed cell line expr.

Fig. S11 | Associating biomarkers of high/low metastatic potential in human cancer cell lines from MetMap and translating them to TCGA patients for different disease types. a) Difference in survival of TCGA cancer patients for which we predict high metastatic potential (top 25%, blue) vs low metastatic potential (bottom 25%, orange) with ML models trained on original cell line expression profiles (left-side plots) or ML models trained on MOBER-transformed cell line expression profiles (right-side plots), segmented by disease type. P-values are derived from the log-rank test, shaded areas indicate 90% of confidence intervals. For all indications, the p-values of the respective log-rank tests are smaller when MOBER-transformed cell line expression values are used, although statistically significant survival differences (p-value <0.05) are observed only for adrenal gland, brain, kidney, liver, pancreatic and soft tissue cancers. b) Spearman's correlation between the predicted metastatic potential of TCGA tumors and their corresponding clinical stages when using ML models trained on original cell line expression profiles (blue) and ML models trained on MOBER-transformed cell line expression profiles (orange). Only indications with available disease stage annotation are shown. We note that the metastatic potential experiments by MetMap rely on limited number of cell lines, thereby making it challenging to extrapolate findings to diverse patient tumors. Yet, our results demonstrate the utility of using the MOBER-transformed gene expression profiles in cell line to patient translational studies.
